# Unique Microbial Characterisation of Oesophageal Squamous Cell Carcinoma Patients with Different Dietary Habits Based on Light Gradient Boosting Machine Learning Classifier

**DOI:** 10.3390/nu17081340

**Published:** 2025-04-14

**Authors:** Shun Liu, Zhifeng Lin, Zhimin Huang, Menglin Yu, Zheng Lin, Zhijian Hu

**Affiliations:** 1 Department of Epidemiology and Health Statistics, School of Public Health, Fujian Medical University, Fuzhou 350122, China; alikeyouyy@foxmail.com (S.L.); linzf@fjmu.edu.cn (Z.L.); huangzhimin@fjmu.edu.cn (Z.H.); menglinyu@fjmu.edu.cn (M.Y.); lz@fjmu.edu.cn (Z.L.); 2 Key Laboratory of Ministry of Education for Gastrointestinal Cancer, Fujian Medical University, Fuzhou 350122, China

**Keywords:** oesophageal squamous cell carcinoma, microorganisms, diet, LightGBM

## Abstract

**Objectives**: The microbiome plays an important role in cancer, but the relationship between dietary habits and the microbiota in oesophageal squamous cell carcinoma (ESCC) is not clear. The aim of this study is to explore the complex relationship between the microbiota in oesophagal tissue and dietary habits in ESCC patients. **Methods**: 173 ESCC patients were included. The method of 16S rRNA sequencing was used to analyze microbial composition and diversity. The LEfSe and Boruta methods were used to screen important microbes, and the LightGBM algorithm distinguished microbes associated with different dietary habits. PICRUST2 and DESeq2 predicted microbial function and screened differential functions. The Pearson test was used to analyze correlations between microbes and functions, and SPARCC microbial symbiotic networks and Cytoscape were used to determine microbial interactions. **Results**: Significant differences in microbial composition were observed among ESCC patients with different dietary habits. LEfSe and Boruta identified three, six, and two significantly different bacteria in the FF/FP, FF/PF, and FF/PP groups, respectively, with AUC values of 0.683, 0.830, and 0.715. PICRUST2 and DESeq2 analysis revealed 3, 11, and 5 significantly different metabolic pathways in each group. *Eubacterium_B sulci* was positively correlated with PWY-6285, PWY-3801, and PWY-5823. PWY-6397 was positively correlated with *undefinded (Fusobacterium_C)*. Microbial network analysis confirmed unique microbial characteristics in different diet groups. **Conclusions**: Different dietary habits lead to alterations in *Eubacterium_B sulci* and *undefinded (Fusobacterium_C)* and related functional pathways.

## 1. Introduction

Oesophageal cancer is a serious malignancy with a rapidly increasing incidence worldwide in recent years. According to the International Agency for Research on Cancer (IARC), the incidence of oesophageal cancer was the 11th highest in the world, with 511,054 new cases, and the mortality rate was the seventh highest, with 445,391 new deaths, as of 6 April 2025 [1]. Oesophageal cancer is histologically classified into two primary types, oesophageal squamous cell carcinoma (ESCC) and oesophageal adenocarcinoma (EAC). Notably, ESCC accounts for approximately 90% of all oesophageal cancer cases and is the major histological subtype of EC in China and worldwide [2]. ESCC is an aggressive form of cancer that grows rapidly and is usually diagnosed at an advanced stage due to the lack of specific early symptoms. As a result, with a five-year survival rate of 15.3 per cent, ESCC has a low survival rate [3]. In recent years, the incidence and mortality rates of ESCC have decreased due to substantial investment in medical resources and advances in medical standards [2]. However, ESCC remains a significant global public health challenge. Therefore, prevention and early diagnosis of ESCC remain of paramount importance.

The pathogenesis of ESCC is closely linked to a variety of factors. Current research has identified several risk factors, including lifestyle behaviours, environmental exposures, and genetic predisposition. For example, smoking, alcohol consumption, micronutrient deficiencies, and exposure to carcinogens have all been implicated in the development of ESCC [4]. Among these factors, dietary habits are particularly noteworthy, as they are considered to be one of the most important contributors to the incidence of ESCC. Dietary habits are inextricably linked to human health, and poor dietary habits are likely to lead to cancer [5]. The impact of poor dietary habits, such as low fruit and vegetable intake and high consumption of pickled vegetables, on the development of ESCC has been the focus of recent research [6]. However, a meta-analysis focusing on Chinese populations found that eating quickly and having a preference for spicy food were the two most significant risk factors among poor dietary habits [7]. Masukume et al. [8] found that long-term consumption of overheated food caused physical damage to the oesophageal mucosa and increased the risk of EC. Song et al. [9] and Ran et al. [10] found that the consumption of fast food, hard food, and hot food may increase the risk of developing oesophageal cancer. However, because most of these studies are based on observational data, and especially because the interactions between different dietary habits are complex and difficult to determine, there are relatively few detailed studies on the determinants of different dietary habits. Based on these findings, this study selected the habits of eating quickly and consuming hot foods as the primary research factors. ESCC patients were divided into four groups with different dietary patterns, with the aim of providing theoretical references for the occurrence, development, and prognosis of ESCC.

A growing number of research suggests that the microbiome plays an integral role in human health and can be modified by dietary practices [11]. Studies have shown that microbial fermentation products, such as short-chain fatty acids (SCFAs), plant secondary metabolites, such as polyphenols and flavonoids, are able to maintain the micro-ecological balance of the gut by selectively promoting the proliferation of beneficial bacteria, thereby preventing inflammation, strengthening the intestinal barrier, and blocking the production of potential pathogens [12,13]. Xiao et al. [14] investigated the impact of hazardous dietary habits on the microbial composition of oesophageal precancerous lesions and found that such habits significantly affected the microbial profiles of affected patients. Specifically, regular alcohol consumption, tea drinking, frequent consumption of hard foods, and fast eating were identified as positively correlated with the occurrence of oesophageal precancerous lesions. Nobel [15] found that patients with a high-fibre diet had a different microbial composition than those with a low-fibre diet. The relative abundance of certain bacteria associated with oesophageal disease was found to be related to fibre intake.

Although studies have been conducted to determine the effects of poor dietary habits on the microbial composition of precancerous oesophagal lesions [14,15], these studies still have many shortcomings: (1) the sample size of the population study is small and the representativeness has certain deficiencies; (2) in China, ESCC is the predominant type of oesophagal cancer, accounting for the vast majority of cases. Despite its prevalence, research on the relationship between dietary factors and the microbial composition of ESCC remains limited, particularly with regard to two important dietary habits: preference for hot and spicy foods and eating quickly. Given this gap in knowledge, the present study aims to investigate the impact of these two dietary habits on the microbiological composition of ESCC. (3) Most of the existing studies have sampled the gut microbiota, while relatively few studies have sampled cancer tissues, limiting the in-depth understanding of ESCC-specific microbial composition and how it relates to dietary habits.

In this study, we collected tissue samples from 173 ESCC patients who underwent oesophagectomy for oesophagal cancer at Fujian Cancer Hospital, First Affiliated Hospital of Fujian Medical University, and Zhangzhou Hospital between 2014 and 2020. Using high-throughput 16S sequencing, we investigated the effects of risky dietary habits on the microbial composition of ESCC. This approach allowed us to elucidate the differences in the oesophageal microbiota under different dietary habits, predict the functions of the oesophageal microbiota, and explore the interactions between microbes. The results of this study not only help to elucidate the mechanisms underlying the association between diet and tumourigenesis but also provide valuable insights for the development of preventive strategies and therapeutic approaches for ESCC.

## 2. Materials and Methods

### 2.1. Studying Population

Our study included 173 ESCC patients who underwent esophagectomy at Fujian Cancer Hospital, First Affiliated Hospital of Fujian Medical University and Zhangzhou Hospital from 2014 to 2020. Patients were consecutively recruited from hospital databases and were divided into four groups based on their eating habits: fast eating and preferring hot food (PP), fast eating but disliking hot food (PF), slow eating but preferring hot food (FP), and slow eating and disliking hot food (FF). Hot food is defined as food or drink that has been cooked and left to stand for less than five minutes; eating speed is defined as fast if eating time is ≤10 min per meal and slow if eating time is >10 min per meal. The recruitment process followed predefined inclusion and exclusion criteria: (1) Undergoing radical oesophagectomy. (2) Pathological diagnosis of squamous cell carcinoma. (3) Patients without other infectious diseases prior to surgery. (4) Not receiving neoadjuvant therapy or adjuvant radiotherapy prior to surgery. (5) Complete clinicopathological and tissue samples. Patients who met all of the following criteria were excluded from this study: (1) Recurrent oesophageal cancer and metastatic tumours. (2) Patients who had undergone oesophagectomy for other reasons. (3) Patients with incomplete prognostic follow-up data. (4) Patients who received antibiotic treatment within one week before surgery, had preoperative blood cell counts indicating infection or had a history of using proton pump inhibitors (PPIs). Finally, 173 eligible patients were included in this retrospective study. Patient basic information and clinical data were extracted from the hospital’s medical records system, ensuring reliability, accuracy, and completeness of the information. Dietary habit data were collected through face-to-face interviews. A standardised questionnaire was used to gather detailed information, and collected data were verified, supplemented, and corrected by trained personnel. Data entry was performed using EpiData 3.1 with double-entry validation. Logical checks and consistency tests were applied to ensure data accuracy and reliability.

### 2.2. Specimens Collection

Oesophageal tumour tissue obtained during surgical resection was snap-frozen in liquid nitrogen and stored at −80 °C until used. All samples were evaluated by pathological haematoxylin and eosin (HE) staining.

### 2.3. DNA Extraction and 16S rRNA Sequencing

Paired tumour and tumour-adjacent samples were collected from ESCC patients immediately after surgical resection in the operating room. The tumour-adjacent samples were taken from an area 3 cm away from the cancerous tissue. The samples were cut into small pieces and placed in autoclaved cryovials, stored in liquid nitrogen for 1 h, and then transferred to a −80 °C refrigerator for storage. All samples were evaluated by pathological haematoxylin and eosin (HE) staining.

### 2.4. Microbial DNA Extraction and Sequencing

Total DNA was extracted using the manufacturer’s sodium dodecyl sulphate (SDS) method, and DNA concentration and purity were monitored on a 1% agarose gel. A blank buffer control was used for each extraction to detect contamination from reagents or other unintended sources. The V3-V4 region of the 16S rRNA gene was amplified using a forward primer (515F: 5′-CCTAYGGGRBGCASCAG-3′) and a reverse primer (806R: 5′-GGACTACNNGGTATCTAAT-3′). All PCR reactions were performed using 15 µL of Phusion^®^ High-Fidelity PCR Master Mix (New England Biolabs, Ipswich, MA, USA). Positive and negative primers were 0.2 μM in diameter and template DNA was approximately 10 ng. Thermal cycling consisted of an initial denaturation at 98 °C for 1 min, denaturation at 98 °C for 10 s, annealing at 50 °C for 30 s, and extension at 72 °C for 30 s, for a total of 30 cycles. The final cycle consisted of 30 cycles of initial denaturation at 98 °C for 10 s, annealing at 50 °C for 30 s, and extension at 72 °C for 30 s. Sequencing was performed on an Illumina Novaseq6000 platform (Illumina, San Diego, CA, USA).

Raw microbial sequencing data were imported into QIIME2-2022.08 [16]; specifically, we used the QIIME2 tools import for 16S rRNA data and generated the demux.qza file, followed by parameterisation based on the demux.qzv file after visualisation of the demux.qza. We then used the DADA2 algorithm [17] for denoising to remove low-quality reads from the sequencing reads, while we filtered the ASV reads that were less than 5% of the sample number, and, for the last retained ASVs, we used the Naïve Bayes classifier (https://docs.qiime2.org/2022.11/tutorials/feature-classifier/, accessed on 11 April 2025) to compare and annotate the filtered amplicon sequence variants (ASVs) and used them to construct subsequent phylogenetic trees.

### 2.5. Diversity Analysis

For both α- and β-diversity analyses, the resampling depth was set to 7000 reads to ensure sufficient reads and sample size. Within-sample diversity (α-diversity) was estimated using several microbial diversity indices (the Shannon index, evenness index, Firth phylogenetic index, and observed ASV index). On the other hand, inter-sample diversity (β-diversity) was analysed by permutational multivariate ANOVA (PERMANOVA) using unweighted and weighted Unifrac distances, Bray–Curtis distance, and Jaccard distance, and by permutational multivariate analysis of dispersion (PERMDISP).

### 2.6. Differential Bacteria and Machine Learning Classifiers

Linear discriminant analysis (LDA) effect size (LEfSe) was used to identify differential bacteria between different diet groups, and the LDA score threshold for discriminant features was 2.5; we stratified the differential bacteria detected by LEfSe by sex and smoking and investigated whether these differential bacteria were influenced by other relevant factors. The Boruta algorithm was used to identify significant differential bacteria in each group, and the doTrace parameter of the Boruta algorithm in this study was set to 2, with the maximum number of iterations set to 100; the relationship between the differential bacteria identified by the Boruta algorithm and clinical factors of ESCC was investigated using the logistic regression method. To further evaluate the ability of the identified significant differential bacteria to discriminate between different diets, the LightGBM machine learning algorithm based on the Gradient Boosted Decision Tree (GBDT) framework was used in this study, and the receiver operating characteristic (ROC) curve of the model was plotted and the area under the curve (AUC) was used to assess the classification performance, and the rank sum test was used for trend analysis of significant differential bacteria in different dietary habit groups.

### 2.7. Functional Analysis and Microbial Symbiotic Networks

We reconstructed communities using the Phylogenetic Investigation of Unobserved States (PICRUSt2) algorithm to predict pathways in the MetaCyc metabolic pathways database (https://metacyc.org/, accessed on 11 April 2025). To identify differences in microbial function between the different dietary habit groups (PP, PF, FP, and FF), we used the DESeq2 differential function detection algorithm. By setting false discovery rate (FDR) < 0.05 and log2-fold change (log2FC) thresholds, we searched for microbial functions that were significantly different when the PP, PF, and FP groups were compared with the FF group. In addition, to explore the correlation relationship between the screened differential functions and significant differential bacteria, we used Pearson correlation analysis to assess the correlation between differential functions and significant differential bacteria.

For bacterial interactions, we used the SPARCC algorithm [18] to assess microbial symbiotic networks and Cytoscape [19] (v3.7.2) to graphically represent symbiotic communities and the relative abundance and strength of correlations of bacteria.

### 2.8. Statistical Analysis

All statistical analyses were performed using R software (version 4.2.3) and EpiData (version 3.1). The chi-squared test was used to compare baseline characteristics of ESCC patients in different dietary habit groups. All significance tests were two-tailed, and a *p* value of less than 0.05 was considered statistically significant. Logistic regression was used for the association analysis of significant bacteria with TNM stage or lymph node metastasis; where High/Low was partitioned based on the median of bacterial relative abundance, we classified Positive and Negative bacteria according to the relative importance of the bacteria detected.

## 3. Results

### 3.1. Characteristics of the Study Population

The study population consisted of 173 ESCC patients who were divided into four groups according to their dietary habits: 53 patients in the PP group, 34 patients in the PF group, 55 patients in the FP group, and 31 patients in the FF group. The differences in gender and smoking status among the ESCC patients enrolled in this study were statistically significant (*p* < 0.05) (Table 1).

### 3.2. Results of the Diversity Analysis

**Figure 1:**
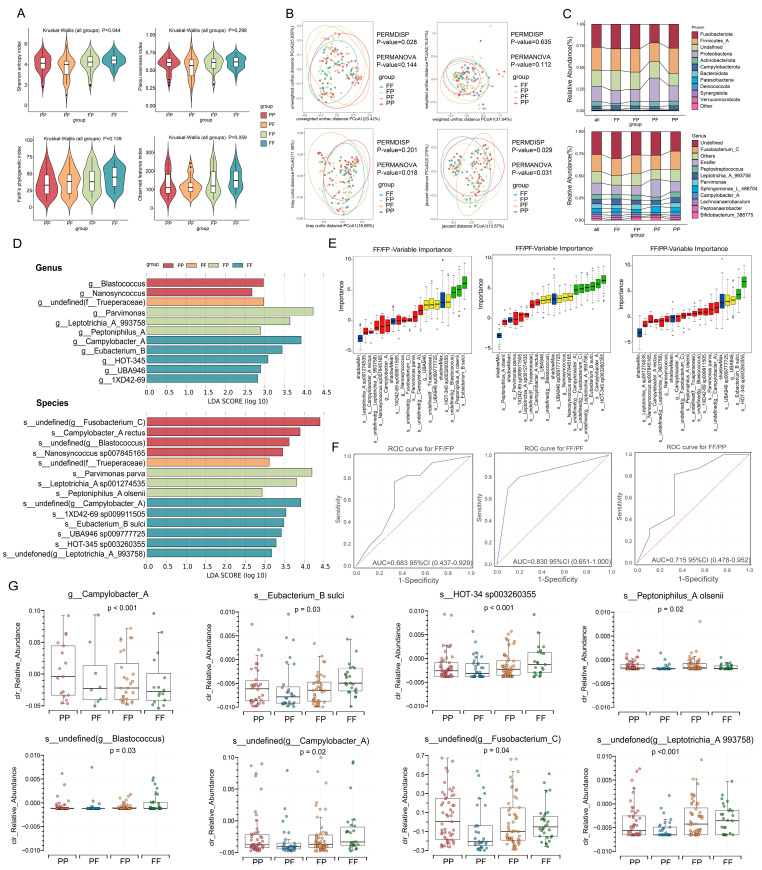
Differential analysis of microbiota of ESCC patients in different dietary habit groups. (**A**) α-diversity violin plots (based on the Shannon diversity index, the Faith phylogenetic diversity index, the observational characterisation index, and the Pielou homogeneity index for the PP, PF, FP, and FF groups; (**B**) β-diversity PCoA plots (based on unweighted UniFrac distance, Jaccard distance, weighted UniFrac distance, and Bray–Curtis distance) for PP, PF, FP, and FF groups; (**C**) microbial composition of ESCC patients in PP, PF, FP, and FF groups at the phylum and genus levels; (**D**) microbial differences in ESCC patients in PP, PF, FP, and FF groups at the genus and species levels; (**E**) box line plots of important differential bacteria in the FF/FP, FF/PF, and FF/PP groups screened by the Boruta algorithm, red: rejected, green: accepted, blue: shadow, yellow: tentative; (**F**) ROC curves of Boruta screening results (FF/FP, FF/PF, and FF/PP groups) by LightGBM machine learning algorithm; (**G**) box line plots of Clr_relative abundance differences among FF, FP, PF, and PP groups for important differential bacteria screened by the Boruta algorithm for FF/FP, FF/PF, and FF/PP groups, red: PP group, blue: PF group, orange: FP group, green: FF group.

In this study, we evaluated the differences in microbial richness and diversity between the four groups PP, PF, FP, and FF. For α-diversity, we assessed it using the Shannon index, which showed significant differences between the different dietary groups (*p* = 0.044, Figure 1A). For β-diversity, the results of Bray–Curtis distance (*p* = 0.018) and Jaccard distance (*p* = 0.031) analyses showed significant differences between the different dietary habit groups (Figure 1B).

### 3.3. Relative Abundance of Species

At the phylum level, the dominant bacteria in the FF, FP, PF, and PP groups were Firmicutes_A, Fusobacteriota, Proteobacteria, and Actinobacteriota, and, at the genus level, *Fusobacterium_C*, *Ensifer*, *Peptostreptococcus*, *Leptotrichia_A_993758*, and *Parvimonas* were the dominant bacterial genera in different dietary habit groups (Figure 1C).

### 3.4. Microbial Differences Among FF, FP, PF, and PP Groups

LEfSe analysis revealed 11 differential bacteria at the genus level, with the three most affected species being *Parvimonas*, *Campylobacter*, and *Leptotrichia_A_993758*. A total of 14 differential bacteria were found at the species level, with the three most affected species being *undefined (Fusobacterium_C)*, *Parvimonas parva,* and *undefined (Campylobacter_A)* (Figure 1D). Tables S1 and S2 show that the differential bacteria screened by LEfSe were not statistically different by sex and whether or not the population smoked.

### 3.5. Unique Bacterial Characteristics of ESCC Patients with Different Diets

The screening results of the Boruta algorithm showed that three, six, and two significant differential bacteria were found in the FF/FP, FF/PF, and FF/PP groups (Figure 1E).

Figure 1F shows the classification performance of the important bacteria. The results indicate that these important bacteria have good classification ability, with an AUC value of 0.683 (95% CI: 0.437–0.929) for FF/FP, an AUC value of 0.830 (95% CI: 0.651–1.000) for FF/PF, and an AUC value of 0.715 (95% CI: 0.478–0.952) for FF/PF. These results suggest that the selected important bacteria have a good ability to distinguish between different dietary habits, and the bacterial differences between ESCC patients in the FF and PF groups are the most significant.

To gain insight into the changes in abundance of the screened key differential bacteria in each group, we further plotted the relative abundance of these eight key differential bacteria after centring in a box line plot (Figure 1G). The results showed that the relative abundance of these eight key differential bacteria was significantly different (*p* < 0.05) between the different dietary habit groups. This finding further confirms the unique characterisation of these key microorganisms under different dietary patterns, suggesting that they may play an important role in the association between diet and microbial community structure. In addition, we found that *Campylobacter_A* was associated with lymph node metastasis (*p* < 0.05) and *undefined (Blactococcus)* was associated with TNM staging (*p* < 0.05) (Table S3).

### 3.6. Unique Bacterial Functional Characteristics in ESCC Patients with Different Dietary Habits

**Figure 2:**
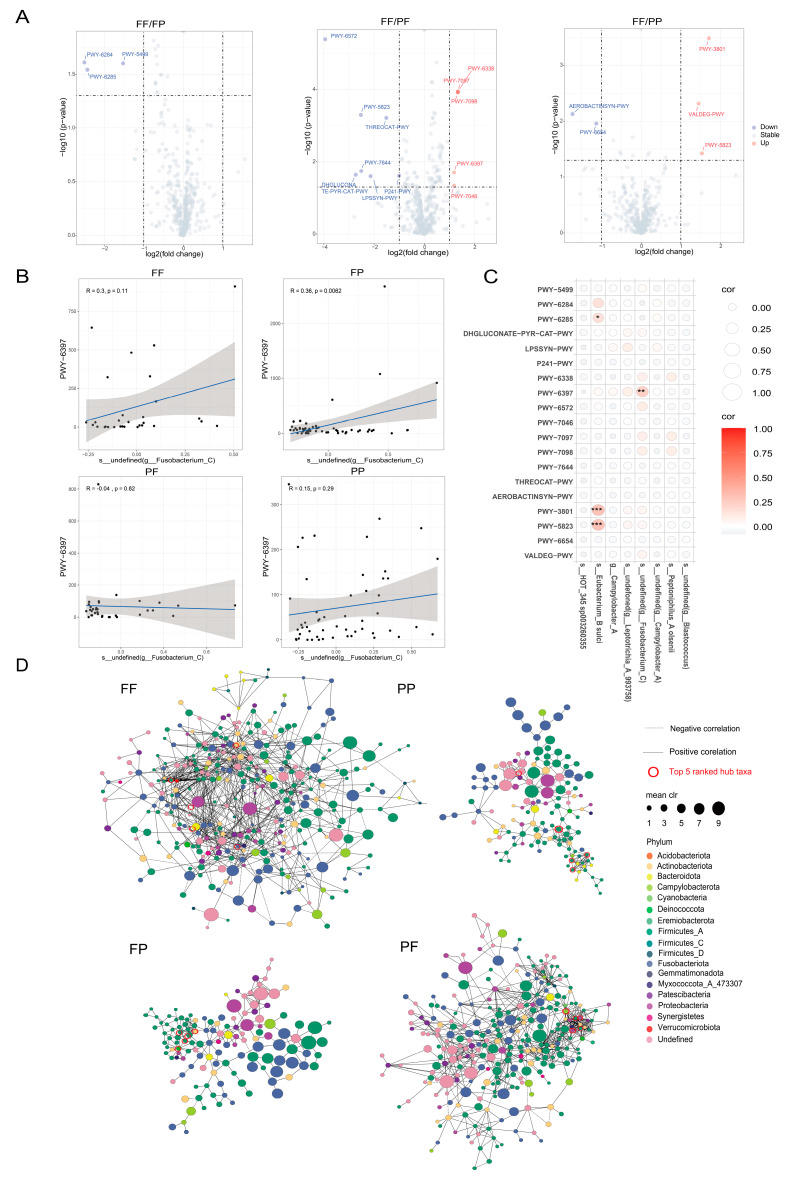
Functional and network analysis of microbiota of ESCC patients with different dietary habits. (**A**) Differential functional volcano plots of FF/FP, FF/PF, and FF/PP groups screened by DESeq2 algorithm; (**B**) scatter plot of correlation between *s__undefined (g__Fusobacterium_C)* and PWY-6397 in FF, FP, PF, and PP groups; (**C**) bubble heat map of correlation analysis of differential function with important bacteria screened by Boruta; (**D**) bacterial network analysis plots for FF, FP, PF, and PP groups.

Metagenomic predictions were performed on the 16S dataset using PICRUSt2. DESeq2 analysis identified differentially expressed pathways in the FF/FP, FF/PF, and FF/PP groups: 3 pathways (PWY-5499, PWY-6284, PWY-6285) in FF/FP, 11 in FF/PF (top five: PWY-6572, PWY-5823, PWY-6338, PWY-7097, PWY-7098), and 5 in FF/PP (AEROBACTINSYN-PWY, VALDEG-PWY, PWY-3801, PWY-5823, PWY-6654) (Figure 2A). Correlation analysis showed that PWY-6285, PWY-3801, and PWY-6397 were positively correlated with *Eubacterium_B sulic* (*p* < 0.05), and PWY-6397 with *undefined (Fusobacterium_C*) (*p* < 0.05) (Figure 2C). This correlation was observed between groups; undefined (Fusobacterium_C) was positively correlated with PWY-6397 in the FF, FP, and PP groups and negatively correlated with PWY-6397 in the PF group, but was not statistically significant (Figure 2B), and the rest of the correlations are shown in Figure S1.

### 3.7. Coexistence Networks of Oesophageal Microbiota with Different Diets

It is well known that, in disease states, the symbiotic relationships between bacteria can change significantly, leading to dysbiosis. Therefore, studying the functional relationship of the oesophagal microbiota may provide a new entry point for understanding the pathophysiological mechanism of disease pathogenesis. We constructed four co-occurrence networks to understand the interactions between oesophageal bacteria in the FF, FP, PF, and PP groups (Figure 2D). We found that the symbiotic relationships between bacteria in ESCC patients with different dietary habits were unique; there were 708 positive correlations and 145 negative correlations in the FF group, and 259 positive correlations and 8 negative correlations in the FP group. There were 761 positive and 63 negative correlations in the PF group and 262 positive and 12 negative correlations in the PP group. In the co-occurrence network of the FF group, the core bacteria are Patescibacteria, Proteobacteria, Firmicutes, Bacteroidota, Synergistetes, Myxococcota_A_473307, and Undefined. In the co-occurrence network of the PP group, the core bacteria are Firmicutes, Proteobacteria, Undefined, Synergistetes, Bacteroidota, and Firmicutes. In the co-occurrence network of the FP group, the core bacteria are Proteobacteria, Undefined, Bacteroidota, Firmicutes, and Myxococcota_A_473307. In the co-occurrence network of the PF group, the core bacteria are Firmicutes, Fusobacteriota, Bacteroidota, Bacteroidota, and Undefined.

## 4. Discussion

Oesophageal cancer includes ESCC and EAC. Among the two pathological types of oesophageal cancer, ESCC dominates in the Chinese population [20]. From 2015 to 2017, the incidence of ESCC in Fujian Province was 1597 cases per 100,000 people, and the mortality rate was 13.71 cases per 100,000 people. Among the causes of death from malignant tumours in Fujian Province, the mortality rate of oesophageal cancer ranked fifth [21]. The symptoms of patients with early oesophagal cancer are not obvious, which makes diagnosis difficult. In addition, the main treatment for ESCC is resection of the oesophageal cancer. Epidemiology shows that the recovery of patients after oesophageal resection is not ideal and the quality of life is poor, which seriously affects the life and health of residents in this area. In this context, it is of great significance to study the possible risk factors of ESCC, especially the influence of multiple risk factors combination on oesophagal microorganisms, so as to provide new ideas for the diagnosis and treatment of ESCC.

Although the aetiology of ESCC is not fully understood, existing studies have shown that dietary habits and microbial factors are closely associated with ESCC [9,14]. The dietary habits of 173 patients with ESCC from different areas of Fujian Province were studied. They were divided into four different dietary habits, fast eating and preferring hot food, fast eating but not preferring hot food, slow eating but preferring hot food, and slow eating but not preferring hot food, and the microbial composition of patients in the four groups and the diversity and richness of microflora between different groups were compared. To explore possible biomarkers of differences in microbiota structure in oesophageal cancer patients with different dietary habits in Fujian Province.

In our analysis, there were 39 women and 134 men with ESCC, similar to the sex ratio reported in the 2017 ESCC epidemiology report [22]. More smoking, drinking, more life pressures, and poor lifestyle habits in men may be the reasons for their higher incidence. We found that there were differences in the microbial diversity of ESCC between different dietary habits, which is consistent with the findings of Hao [23]. During tumour development, differences in daily life and rest would lead to different internal microenvironments, and changes in the microenvironment would lead to changes in microbial structure, and the abundance of a microbial population would increase with disease progression. Along with the decrease in the relative abundance of another microbial population, the microorganisms with higher biomass have traditionally been thought to play a major role in cancer progression, but some studies have reported that microorganisms with lower biomass can also promote cancer development [24,25], which has broadened our view of the relationship between microorganisms and cancer to some extent.

The study by Li [26] showed that Firmicutes and Bacteroidetes were more abundant and significantly different in ESCC tissue samples compared to normal controls, which was similar to the microbial composition of the four groups with different dietary habits in this study. This suggests that Firmicutes, Clostriobacteria, and Bacteroidetes may have a potential relationship with the occurrence and development of ESCC.

At the genus level, we show the species composition of patients with different dietary habits. There was no significant difference in the composition of the top five dominant bacteria among the different groups, but the results of the α diversity calculation showed that there were differences in oesophageal microbial diversity among patients with different dietary habits in the four groups according to the Shannon index. Prakash [27] evaluated the effect of hot food on oral bacterial growth and the results showed that the bacterial growth of volunteers who drank hot tea was significantly reduced (*p* < 0.001). Another study of ESCC in Northwest China [28] showed that hot drinks increased mechanical damage to the oesophagus. This may also be the reason for the reduction in oesophageal microbial diversity caused by hot food. Case-control [29] studies have shown that fast food is positively associated with the risk of developing oesophageal cancer. However, this study did not find that these two factors had a significant promoting effect on ESCC.

The Boruta and LightGBM algorithms were used for in-depth analysis of the gut microbial communities in four groups with different poor dietary habits. The ROC curve analysis of the LightGBM model showed AUC values of 0.683, 0.830, and 0.715 for the FF/FP, FF/PF, and FF/PP groups, respectively, indicating that the differential bacteria screened by the Boruta algorithm were able to better represent the differences in different dietary habits, which further confirmed the association between poor dietary habits and changes in microbial abundance in oesophageal tissues. In our study, we hypothesised that different diets may affect ESCC by altering the relative abundance of bacteria, with certain bacteria being more affected, which we termed important bacteria, and that imbalances in these important bacteria may play a more dominant role in the influence of dietary factors on ESCC. Imbalances in these key bacteria may be critical in determining how dietary factors affect ESCC. Furthermore, different diets are associated with different key bacteria, suggesting that different dietary patterns may have unique carcinogenic properties.

In this study, we identified both common and unique important differential bacteria across different dietary groups. *Eubacterium_B_sulci*, a member of the normal human gut flora, was detected in the significant differential bacteria screens of all three groups [30]. As shown in Figure 1G, the relative abundance of *Eubacterium_B_sulci* gradually decreased with the increase in poor dietary habits, suggesting that *Eubacterium_B_sulci* may be a protective factor for ESCC. Consistent with our findings, previous studies have shown that Eubacterium is involved in intestinal food fermentation, metabolism of SCFAs, cholesterol, and bile acids, and has anti-inflammatory properties [30]. *Campylobacter_A* belongs to the genus *Campylobacter*. The study by He [31] showed that *Campylobacter* is enriched in primary tumours of patients with metastatic colorectal cancer and promotes cancer metastasis by producing cytolethal dilatation toxin (CDT). In our study, the relative abundance of *Campylobacter_A* was lowest in the FF group and highest in the PP group. This finding is consistent with previous research and suggests that *Campylobacter_A* may be a risk factor for ESCC.

This study also investigated the MetaCyc pathway and microbial interaction network in ESCC patients. The four groups of ESCC patients with different diets had different MetaCyc pathways, suggesting that the development of ESCC involves multiple functional and metabolic changes and that cancer progression is the result of multiple interactions. In this study, we performed an in-depth analysis of the potential mechanisms of action of eight important differential bacteria. By analysing the correlations between 19 differential pathways and the relative abundance of these differential bacteria, we found that *Eubacterium_B_sulci* showed significant positive correlations with three pathways. In this study, *Eubacterium_B_sulci* has a significant positive correlation with three metabolic pathways, and PWY-5823 (a metabolic regulatory pathway related to p53) has been confirmed to have an inhibitory effect on the occurrence and development of cancer. Specifically, wild-type p53 can restrict the growth of tumour cells by inhibiting glycolysis, promoting oxidative phosphorylation, and inhibiting the pentose phosphate pathway and fatty acid synthesis; in addition, p53 can regulate target genes such as TIGAR (glycolytic inhibitor) and GLS2 (glutaminase) and reduce reactive oxygen species (ROS) levels and inhibit anabolism [32,33]. Based on the above results, in this study, we speculated that the preference for hot food and fast eating habits may reduce the relative abundance of *Eubacterium_B_sulci* in the oesophagus and then weaken the function of PWY-5823. The risk of oesophagal squamous cell carcinoma (ESCC) may increase with the reduction in PWY-5823’s cancer-suppressing function. In addition, *undefined (Fusobacterium_C)* showed a significant positive correlation with PWY-6397, and this correlation was differentiated among the four groups with different dietary habits; *undefined (Fusobacterium_C)* showed a negative correlation with PWY-6397 in the PF group and a positive correlation in the remaining three groups, further suggesting that different dietary habits do not have the same carcinogenic profile. This uniqueness was also reflected in the microbial interaction network, with the FF group having a denser microbial interaction network, while the remaining three groups had a significantly reduced microbial interaction network, and it was hypothesised that the reason could be the reduction in certain bacteria due to poor dietary habits, which was consistent with previous results. At the same time, it was found that, in the four groups of ESCC patients with different dietary conditions, the microorganisms mainly promoted each other, which provided a certain idea for early prevention of the disease. We also found a functional pathway related to REDOX in the FF group, which is similar to the study by Chen et al. [34].

In conclusion, dietary habits are extremely important factors influencing the occurrence and development of ESCC and altering the structure of the oesophageal flora. In this study, we compared the structural differences of microorganisms by α-diversity and β-diversity and screened microorganisms associated with different poor dietary habits by linear discriminant analysis. MetaCyc pathways of microorganisms were explored by PICRUSt2 analysis, and, finally, the interaction network between microorganisms was explored. This study provides evidence at the microbial level of the influence of dietary habits on the development of ESCC, but there are still shortcomings: this study is at the level of exploration and discovery, and it remains to be explored whether bacteria related to eating habits can really promote the development of ESCC. Future studies will mainly focus on the following parts: conduct experiments to demonstrate the correlation between the selected bacteria associated with eating habits and ESCC.

## 5. Conclusions

In this study, we investigated the microbiota of ESCC patients associated with different dietary habits, and our results suggest that different dietary habits may lead to alterations in *Eubacterium_B sulci* and *undefinded (Fusobacterium_C)*, and, consequently, in PWY-6285, PWY-3801, PWY- 5823, and PWY-6397 functional pathway expression.

## Figures and Tables

**Table 1:** Characteristics of ESCC patients with different dietary habits.

Variables	All Patients n (%)	FF n (%)	FP n (%)	PF n (%)	PP n (%)	*χ* ^2^	*p* Value
Gender							
Female	39 (22.5)	11 (35.5)	17 (30.9)	2 (5.9)	9 (17.0)	11.522	0.009
Male	134 (77.5)	20 (64.5)	38 (69.1)	32 (94.1)	44 (83.0)		
Age							
>60	92 (53.2)	19 (61.3)	27 (49.1)	17 (50.0)	29 (54.7)	1.377	0.711
≤60	81 (46.8)	12 (6.9)	28 (50.9)	17 (50.0)	24 (45.7)		
T stage							
I/II	60 (34.7)	8 (25.8)	23 (41.8)	12 (35.3)	17 (32.1)	2.479	0.479
III/IV	113 (65.3)	23 (74.2)	32 (58.2)	22 (64.7)	36 (67.9)		
Lymph node metastasis							
No	66 (38.2)	9 (29.0)	26 (47.3)	11 (32.4)	20 (37.7)	3.520	0.318
Yes	107 (61.8)	22 (71.0)	29 (52.7)	23 (67.6)	33 (62.3)		
Tumor location							
Lower	71 (41.0)	11 (35.5)	23 (41.8)	12 (35.3)	25 (47.2)	1.696	0.638
Middle/Upper	102 (41.0)	20 (64.5)	32 (58.2)	22 (64.7)	28 (52.8)		
Tea consumption							
No	49 (28.3)	12 (38.7)	20 (36.4)	7 (20.6)	10 (18.9)	6.735	0.081
Yes	124 (71.7)	19 (61.3)	35 (63.6)	27 (79.4)	43 (81.1)		
Alcohol consumption							
No	76 (43.9)	15 (48.4)	25 (45.5)	15 (44.1)	21 (39.6)	0.702	0.873
Yes	97 (56.1)	16 (51.6)	30 (54.5)	19 (55.9)	32 (60.4)		
Smoke							
No	53 (30.6)	13 (41.9)	21 (38.2)	5 (14.7)	14 (26.4)	7.841	0.049
Yes	120 (69.4)	18 (58.1)	34 (61.8)	29 (85.3)	39 (73.6)		
Region							
Minnan	110 (63.6)	17 (54.8)	34 (61.8)	20 (58.8)	39 (73.6)	3.720	0.293
Others	63 (36.4)	14 (45.2)	21 (38.2)	14 (41.2)	14 (26.4)		
Sampling season							
Winter/spring	91 (52.6)	15 (48.4)	25 (45.5)	20 (58.8)	31 (58.5)	2.613	0.455
Summer/autumn	82 (47.4)	16 (51.6)	30 (54.5)	14 (41.2)	22 (41.5)		

## Data Availability

The original contributions presented in this study are included in the article/Supplementary Material. Further inquiries can be directed to the corresponding author.

## Supplementary Materials

The following supporting information can be downloaded at https://www.mdpi.com/article/10.3390/nu17081340/s1, Figure S1: Scatterplot of the correlation of *s__Eubacterium_B_sulci* with PWY-3801, PWY-5823, and PWY-6285 in FF, FP, PF, and PP groups. A, FP group; B, FP group; C, PF group; D, PP group; Table S1: Rank-sum test for differential bacteria retained by the LEfSe method in sex-disaggregated populations; Table S2: Rank-sum test for different bacteria retained by the LEfSe method in a population disaggregated by smoking or not smoking; Table S3: Logistic regression analysis of important species with TNM staging and lymph node metastasis in ESCC.
