# Supplementary material for: Unique Microbial Characterisation of Oesophageal Squamous Cell Carcinoma Patients with Different Dietary Habits Based on Light Gradient Boosting Machine Learning Classifier
Source: Nutrients. 2025 Apr 14;17(8):1340. doi: 10.3390/nu17081340 (PMC12030675; doi:10.3390/nu17081340)
Supplement: Supplementary file 1 [file nutrients-17-01340-s001.zip › nutrients-3584095-supplementary.pdf]

Table S1 Rank-sum test for differential bacteria retained by the LEfSe method in sex-disaggregated populations

Bacteria, Median (Q1-Q3)	Overall n=173	Male n=134	Female n=39	p
g__Nanosyncoccus	0.000 (0.000, <0.001)	0.000 (0.000, <0.001)	0.000 (0.000, <0.001)	0.391
g__UBA946	0.000 (0.000, <0.001)	0.000 (0.000, <0.001)	0.000 (0.000, 0.000)	0.124
g__Campylobacter_A	0.008 (0.004, 0.026)	0.009 (0.004, 0.026)	0.008 (0.004, 0.025)	0.657
s__HOT-345 sp003260355	0.001 (<0.001, 0.001)	0.001 (<0.001, 0.001)	0.001 (<0.001, 0.001)	0.838
s__Leptotrichia_A sp001274535	0.003 (0.001, 0.005)	0.003 (0.001, 0.005)	0.003 (0.001, 0.007)	0.35
s__Parvimonas parva	0.029 (0.012, 0.062)	0.031 (0.011, 0.063)	0.022 (0.013, 0.047)	0.486
s__Eubacterium_B sulci	0.002 (0.001, 0.003)	0.002 (0.001, 0.004)	0.001 (0.001, 0.002)	0.285
s__Peptoniphilus_A olsenii	<0.001 (0.000, <0.001)	<0.001 (0.000, <0.001)	0.000 (0.000, <0.001)	0.441
s__Nanosyncoccus sp007845165	0.000 (0.000, <0.001)	0.000 (0.000, <0.001)	0.000 (0.000, 0.000)	0.4
s__1XD42-69 sp009911505	0.000 (0.000, <0.001)	0.000 (0.000, <0.001)	0.000 (0.000, 0.000)	0.051
s__UBA946 sp009777725	0.000 (0.000, <0.001)	0.000 (0.000, <0.001)	0.000 (0.000, <0.001)	0.536
s__Campylobacter_A rectus	0.002 (0.001, 0.009)	0.003 (0.001, 0.009)	0.002 (0.001, 0.008)	0.472
s__undefoned(g__Leptotrichia_A_993758)	0.001 (0.000, 0.002)	0.001 (0.000, 0.002)	0.001 (0.000, 0.002)	0.918
s__undefined(g__Fusobacterium_C)	0.091 (0.044, 0.211)	0.090 (0.044, 0.211)	0.091 (0.047, 0.176)	0.814
s__undefined(f__Trueperaceae)	0.000 (0.000, 0.001)	0.000 (0.000, <0.001)	0.000 (0.000, 0.001)	0.546
s__undefined(g__Blastococcus)	0.000 (0.000, <0.001)	0.000 (0.000, <0.001)	0.000 (0.000, 0.000)	0.08
s__undefined(g__Campylobacter_A)	0.004 (0.002, 0.010)	0.004 (0.002, 0.010)	0.003 (0.002, 0.011)	0.666

g__Blastococcus, g__undefined(f__Trueperaceae) g__Parvimonas, g__Leptotrichia_A_993758, g__Peptoniphilus_A, g__Eubacterium_B, g__HOT-345, g__1XD42-69 with, s__undefined(g__Blastococcus), s__undefined(f__Trueperaceae), s__Parvimonas parva, s__undefoned(g__Leptotrichia __A_993758), s__Peptoniphilus_A olsenii, s__Eubacterium_B sulci, s__HOT-345 sp003260355, s__1XD42-69 sp009911505 are classifications of the same species at the genus and species level

Table S2 Rank-sum test for different bacteria retained by the LEfSe method in a population disaggregated by smoking or not smoking

Bacteria, Median (Q1-Q3)	Overall n=173	no-smoke n=53	Smoke n=120	p
g__Nanosyncoccus	0.000 (0.000, <0.001)	0.000 (0.000, <0.001)	0.000 (0.000, <0.001)	0.805
g__UBA946	0.000 (0.000, <0.001)	0.000 (0.000, <0.001)	0.000 (0.000, <0.001)	0.841
g__Campylobacter_A	0.008 (0.004, 0.026)	0.008 (0.003, 0.026)	0.009 (0.004, 0.025)	0.611
s__HOT-345 sp003260355	0.001 (<0.001, 0.001)	<0.001 (<0.001, 0.001)	0.001 (<0.001, 0.002)	0.354
s__Leptotrichia_A sp001274535	0.003 (0.001, 0.005)	0.003 (0.001, 0.006)	0.003 (0.001, 0.005)	0.264
s__Parvimonas parva	0.029 (0.012, 0.062)	0.028 (0.014, 0.055)	0.031 (0.012, 0.064)	0.851
s__Eubacterium_B sulci	0.002 (0.001, 0.003)	0.002 (<0.001, 0.003)	0.001 (0.001, 0.004)	0.818
s__Peptoniphilus_A olsenii	<0.001 (0.000, <0.001)	<0.001 (0.000, <0.001)	<0.001 (0.000, <0.001)	0.999
s__Nanosyncoccus sp007845165	0.000 (0.000, <0.001)	0.000 (0.000, <0.001)	0.000 (0.000, <0.001)	0.423
s__1XD42-69 sp009911505	0.000 (0.000, <0.001)	0.000 (0.000, 0.000)	0.000 (0.000, <0.001)	0.839
s__UBA946 sp009777725	0.000 (0.000, <0.001)	0.000 (0.000, <0.001)	0.000 (0.000, <0.001)	0.911
s__Campylobacter_A rectus	0.002 (0.001, 0.009)	0.003 (0.001, 0.011)	0.002 (0.001, 0.008)	0.335
s__undefoned(g__Leptotrichia_A_993758)	0.001 (0.000, 0.002)	0.001 (0.000, 0.002)	<0.001 (0.000, 0.002)	0.184
s__undefined(g__Fusobacterium_C)	0.091 (0.044, 0.211)	0.088 (0.051, 0.200)	0.093 (0.043, 0.211)	0.87
s__undefined(f__Trueperaceae)	0.000 (0.000, 0.001)	0.000 (0.000, <0.001)	0.000 (0.000, 0.001)	0.139
s__undefined(g__Blastococcus)	0.000 (0.000, <0.001)	0.000 (0.000, <0.001)	0.000 (0.000, <0.001)	0.683
s__undefined(g__Campylobacter_A)	0.004 (0.002, 0.010)	0.003 (0.001, 0.011)	0.004 (0.002, 0.010)	0.627

g__Blastococcus, g__undefined(f__Trueperaceae) g__Parvimonas, g__Leptotrichia_A_993758, g__Peptoniphilus_A, g__Eubacterium_B, g__HOT-345, g__1XD42-69 with, s__undefined(g__Blastococcus), s__undefined(f__Trueperaceae), s__Parvimonas parva, s__undefoned(g__Leptotrichia __A_993758), s__Peptoniphilus_A olsenii, s__Eubacterium_B sulci, s__HOT-345 sp003260355, s__1XD42-69 sp009911505 are classifications of the same species at the genus and species level

Table S3 Logistic regression analysis of important species with TNM staging and lymph node metastasis in esophageal squamous carcinoma

Bacteria species	TNM		lymphatic node transfer	
	OR (95%CI)	P	OR (95%CI)	P
g__Campylobacter_A				
High	1.00 (Reference)		1.00 (Reference)	
Low	1.86 (0.98 - 3.52)	0.056	2.61 (1.38 - 4.95)	0.003
s__HOT-345 sp003260355				
Negative	1.00 (Reference)		1.00 (Reference)	
Postive	1.33 (0.64 - 2.76)	0.439	1.13 (0.55 - 2.33)	0.742
s__Eubacterium_B sulci				
Negative	1.00 (Reference)		1.00 (Reference)	
Postive	1.72 (0.59 - 4.99)	0.321	1.49 (0.51 - 4.33)	0.46
s__undefined(g__Leptotrichia_A_993758)				
Negative	1.00 (Reference)		1.00 (Reference)	
Postive	1.64 (0.85 - 3.15)	0.138	0.97 (0.51 - 1.85)	0.922
s__undefined(g__Campylobacter_A)				
Negative	1.00 (Reference)		1.00 (Reference)	
Postive	1.36 (0.41 - 4.50)	0.61	0.81 (0.23 - 2.81)	0.742
s__Peptoniphilus_A olsenii				
Negative	1.00 (Reference)		1.00 (Reference)	
Postive	1.23 (0.66 - 2.31)	0.513	0.87 (0.47 - 1.62)	0.667
s__undefined(g__Blastococcus)				
Negative	1.00 (Reference)		1.00 (Reference)	
Postive	4.90 (2.04 - 11.75)	<0.001	1.49 (0.74 - 2.98)	0.261
s__undefined(g__Fusobacterium_)C				
High	1.00 (Reference)		1.00 (Reference)	
Low	1.11 (0.59 - 2.07)	0.749	1.05 (0.57 - 1.95)	0.875

OR: Odds Ratio, CI: Confidence Interval

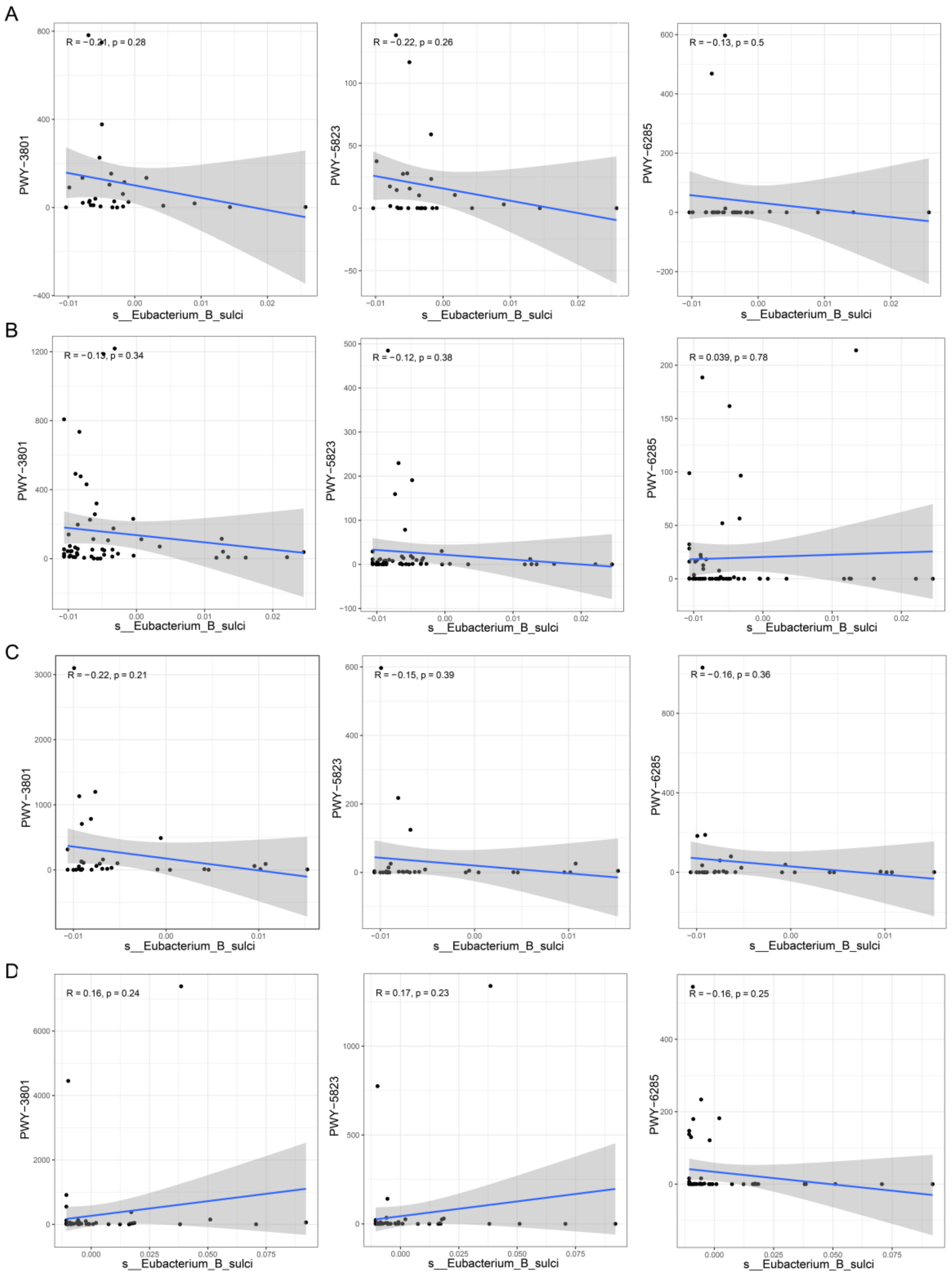

Figure S1 Scatterplot of the correlation of *s__Eubacterium_B_sulci* with PWY-3801, PWY-5823 and PWY-6285 in FF, FP, PF and PP groups. A, FP group; B, FP group; C, PF group; D, PP group.
